# Telomerase Impinges on the Cellular Response to Oxidative Stress Through Mitochondrial ROS-Mediated Regulation of Autophagy

**DOI:** 10.3390/ijms20061509

**Published:** 2019-03-26

**Authors:** Paula D. Green, Nilesh K. Sharma, Janine Hertzog Santos

**Affiliations:** 1 Department of Pharmacology and Physiology, New Jersey Medical School, Rutgers The State University of New Jersey, 185 South Orange Avenue, Medical Sciences Building, Newark, NJ 07103, USA; pauladgreen@gmail.com (P.D.G.); nilesh.sharma@dpu.edu.in (N.K.S.); 2 Department of Molecular Biology and Genetics, Dr. D.Y. Patil Vidyapeeth’s, Dr. D.Y. Patil Biotechnology and Bioinformatics Institute, Mumbai- Bangalore Highway, Tathawade, Pune 411033, India; 3 Current address: National Toxicology Program, National Institute of Environmental Health Sciences (NIEHS), National Institutes of Health, 111 TW Alexander Drive Blg 101, Durham, NC 27709, USA

**Keywords:** telomerase, mitochondria, oxidative stress, autophagy

## Abstract

Telomerase has cellular functions beyond telomere stabilization, including a role in mitochondria. The function of the catalytic component—TERT—in mitochondria is still unknown, but it seems to play a role in the response to oxidative stress. Here, we interrogated the role of the subcellular localization of TERT to the response to hydrogen peroxide (H_2_O_2_) treatment. Using normal human fibroblasts (NHF) expressing non-tagged wild type (WT) human TERT (hTERT) or nuclear localization and function (_nuc_hTERT), a mutant that we previously described as being competent in telomere elongation, while not being able to localize to mitochondria, we found the differential activation of autophagy as a function of hTERT’s subcellular localization. Specifically, we found that only cells expressing the mutant had significant increases in autophagy markers as a response to H_2_O_2_ challenge. Either the reintroduction of the mitochondrial pool of hTERT or the expression of mitochondrially-targeted catalase in mutant cells blunted the autophagic response under oxidative stress. Interestingly, autophagy activation was also associated with decreased levels of mitochondrial DNA damage. Taken together, these results suggest that the loss of hTERT in mitochondria initiates a signaling cascade that allows for cells to adapt to and cope with the lack of mitochondrial telomerase. Such effects also influence the cellular response to oxidative damage.

## 1. Introduction

Telomerase is a ribonucleoprotein that is primarily responsible for telomere maintenance that is a common target in cancer therapy. Since the catalytic component of telomerase (TERT) is also mitochondrial [1,2,3,4,5], it is anticipated that the inhibition of telomerase will not only affect telomere biology, but also mitochondrial function. Consistent with this view, we previously showed in a cell culture model that the expression of mutant human TERT (hTERT) that is unable to enter mitochondria, while maintaining its nuclear localization and function (_nuc_hTERT), led to decreased mitochondrial DNA (mtDNA) integrity, increased mitochondrial reactive oxygen species (mtROS), and altered mitochondrial ultrastructure [3]. In a TERT (mTERT) knockout (KO) mouse model, systemic mitochondrial dysfunction prior to telomere defects was also observed [6]. Later, it was also shown, in a cancer-prone mouse model, that mTERT extinction resulted in mitochondrial dysfunction and short telomeres. While the reinstatement of telomere maintenance by recombination (ALT) allowed for the re-emergence of resistant tumors, it did not completely alleviate mitochondria dysfunction [7], indicating that TERT has an impact on mitochondria that cannot be compensated by telomere stabilization. 

Telomerase has also been implicated in the cellular response to genotoxic stress. Still unresolved are opposing findings that are related to the protection or promotion of DNA damage and cell death, as induced by hTERT, particularly as it relates to oxidative stress [1,2,8,9,10,11]. In this context, our own findings were counterintuitive, as, while we showed that cells expressing _nuc_hTERT had dysfunctional mitochondria at baseline [3], they were also highly resistant to oxidative damage. Instead, cells expressing the wild type (WT) protein were sensitive [2]. A detailed analysis of the electron microscopy (EM) images also revealed an increased number of autophagosomes in _nuc_hTERT [3], which led us to hypothesize that autophagy may play a role in the resistance of mutant cells to oxidative damage. In this work, we capitalized our previously characterized cells expressing WT hTERT- or _nuc_hTERT [1,2] to show that the subcellular localization of hTERT affects the sensitivity to hydrogen peroxide (H_2_O_2_) treatment, at least in part, by differentially regulating autophagy. 

## 2. Results

**Figure 1:**
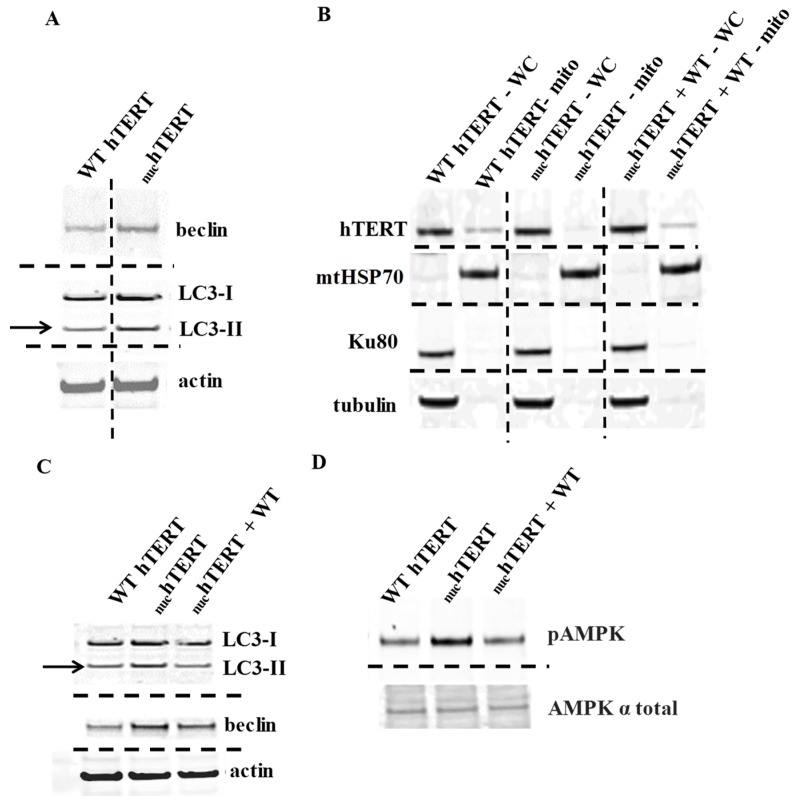
Autophagy and AMP-activated protein kinase (AMPK) are upregulated in nuclear localization and function (_nuc_hTERT) under basal conditions due to lack of the mitochondrial localization of telomerase. (**A** and **C**) Expression levels of autophagy markers LC3-II and beclin in untreated cells. The position of LC3-II is indicated by thin black arrow, actin was used as the loading control. (**B**) Representative immunoblots (N = 3) of protein lysates from whole cell extracts and mitochondrial extracts from each corresponding cell line—wild type human TERT (WT hTERT), _nuc_hTERT, and _nuc_hTERT + WT. Tubulin was used to check for cytoplasmic contamination and Ku80 was used to check for nuclear contamination; mtHSP70 probed enrichment of the mitochondrial fraction. (**D**) Representative (N = 5) immunoblot analysis of phosphor-active AMPK in untreated normal human fibroblasts (NHF) WT hTERT, _nuc_hTERT, and _nuc_hTERT + WT cells. Total AMPK was used as loading control.

AMP-activated protein kinase (AMPK) and autophagy are robustly activated in _nuc_hTERT—but not in WT hTERT—expressing cells upon oxidative stress. As autophagy can enable cells to maintain homeostatic functions in response to cellular stresses, including mitochondrial dysfunction [12], we started by evaluating the autophagy markers beclin and LC3-II by Western blots in normal human fibroblasts (NHF) expressing WT or _nuc_hTERT. At baseline, the levels of beclin and LC3-II were slightly increased in the mutant-expressing (Figure 1A), which is in agreement with the EM data [3]. To determine the contribution of organellar hTERT to these effects, we re-instated the mitochondrial pool of the protein by infecting the _nuc_hTERT-expressing cells (puromycin-resistant), with full length hTERT being expressed in a retroviral vector (hTERTpLXIN) carrying a G418 cassette. Double transfectants were selected based on puromycin and G418 resistance, and Western blots gauged the mitochondrial pool of hTERT (Figure 1B). We then probed autophagy markers and found that the levels of LC3-II and beclin were blunted when hTERT was reinstated in the mutant (Figure 1C). AMP-activated protein kinase (AMPK) is a master metabolic sensor that can regulate autophagy through its inhibition of mTOR (mammalian target of rapamycin) [13]. AMPK is activated through phosphorylation at threonine (Thr) 172 in response to changes in the AMP:ATP ratio and by ROS [14]. The phosphorylation of AMPK at Thr172 was increased in _nuc_hTERT-expressing cells when compared to WT controls and it was also blunted in double transfectants (Figure 1D). Thus, we conclude that the presence of hTERT only in the nucleus is associated with the activation of autophagy. 

**Figure 2:**
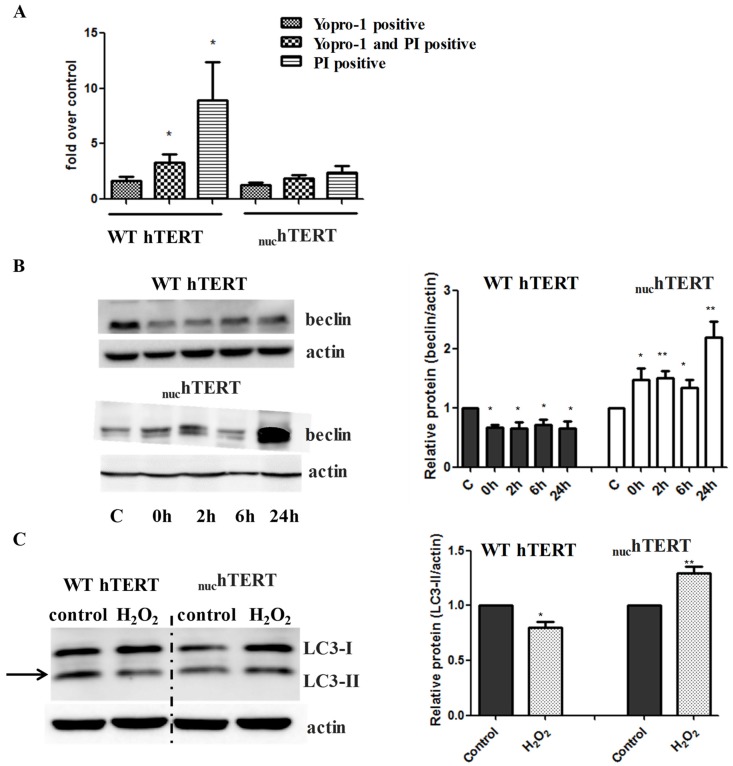

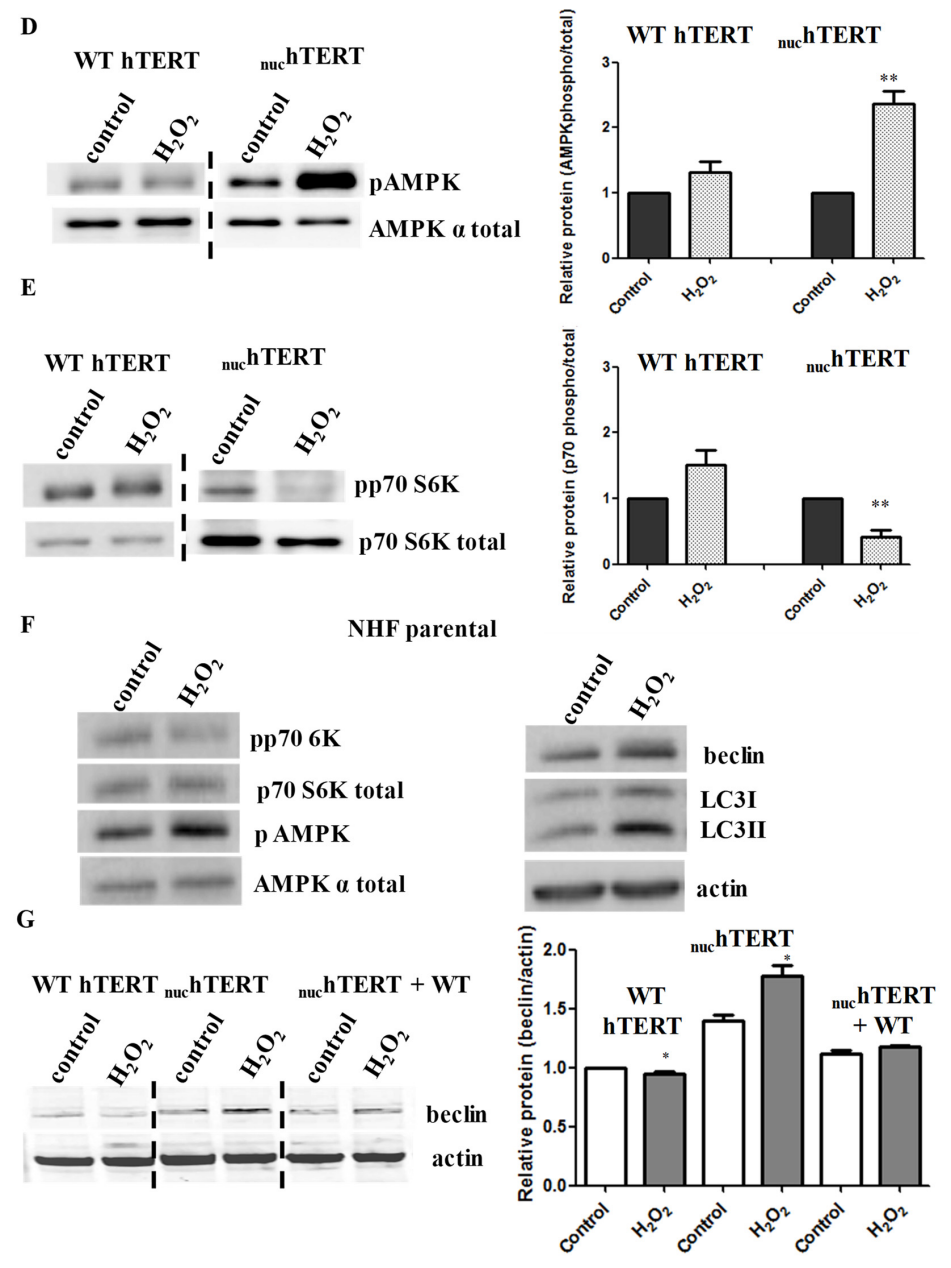

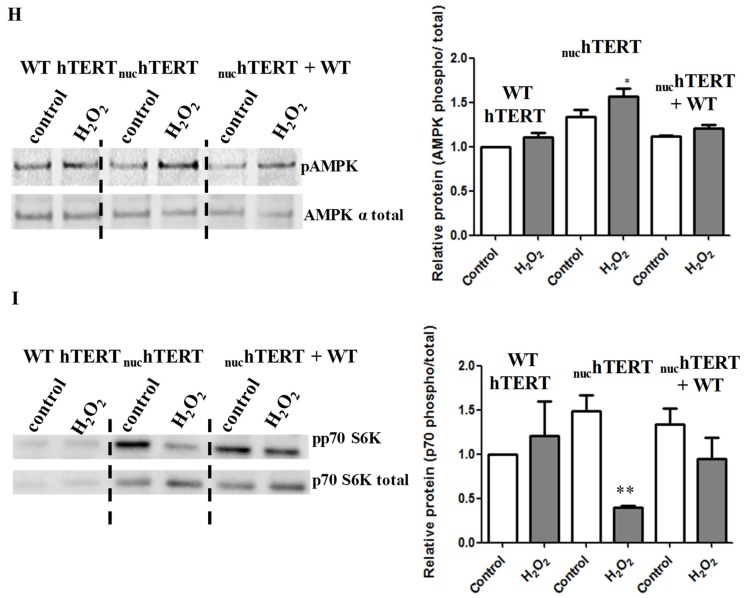
Autophagy is upregulated upon oxidative stress but only when hTERT does not enter mitochondria. (**A**) WT hTERT and _nuc_hTERT were submitted to 60 min of 200 µM H_2_O_2_ treatment and allowed to recover in conditioned medium for 24 h. The cells were then harvested and assayed with Yopro-1 and propidium iodide to monitor apoptosis (YoPro-1) and cell death (propidium iodide) using flow cytometry. Graphs depict the mean of at least three biological experiments ± SEM. Student *t*-test was performed by comparing each corresponding cell death marker to its representative control. *p* value < 0.05 *. (**B**) WT hTERT and _nuc_hTERT cells were subjected to 200 µM of H_2_O_2_ for 60 min (0 h), and allowed to recover at two, six, and 24 h. After the removal of H_2_O_2_ the cells were cultured in conditioned medium (medium in which cells had grown overnight containing cells released-growth factors) for the two, six, and 24 h recovery time points. Levels of beclin were normalized to actin. Graph represents the average of eight independent experiments ± SEM. Student *t*-test was performed by comparing each treated sample — 60 min H_2_O_2_ and the recovery time points to its respective control (untreated cells). Statistical significance was determined at *p* value < 0.05 * and 0.01 **. (C) LC3-II levels were assayed by immunoblots following H_2_O_2_ treatment in WT hTERT and _nuc_hTERT expressing cells. The levels of LC3-II were normalized to actin. Graph shows average of 8 independent experiments ± SEM. Student *t*-test was performed by comparing each 60-minute H_2_O_2_ treatment to its respective untreated control. Statistical significance was *p* value < 0.01 **. (**D**–**E**) Representative Western blot analysis of extracts from cells (WT hTERT and _nuc_hTERT) untreated (control) and treated (200 µM H_2_O_2_ for 60 min) against phospho AMPKα (Thr172), AMPKα total, phospho p70S6K (Thr389) and p70S6K total antibodies**.** Graphs show average data from four independent experiments ± SEM. Phosphorylated AMPKα (Thr172) density was normalized to total AMPKα. Phosphorylated p70S6K density was normalized to total p70S6K. Student’s *t*-test was used to evaluate statistical significance. Student *t*-test compared each H_2_O_2_ treated hTERT expressing cells to its representative untreated control. Statistical significance was determined at a *p* value < 0.05 * and 0.01 **. (**F**) NHF parental cells were submitted to the same H_2_O_2_ treatment as above and autophagy markers analyzed. (**G**–**I**) Representative Western blot analysis of extracts from cells (WT hTERT, _nuc_hTERT, and _nuc_hTERT + WT) untreated (control) and treated (200 µM H_2_O_2_ for 60 min) against beclin, phospho AMPKα (Thr172), AMPKα total, phospho p70S6K (Thr389), and p70S6K total antibody. Actin was also included as loading control for beclin. Graphs show average data from three experiments ± SEM. Student’s *t*-test was used to evaluate statistical significance and then compared each untreated (control) to its respective H_2_O_2_ treatment. Statistical significance was determined at a *p* value < 0.05 * and 0.01 **.

Next, we tested the degree to which autophagy was activated in cells that were exposed to H_2_O_2_. To this end, the same number of WT or _nuc_hTERT cells were plated, and 16 h later the cells were exposed to 200 µM H_2_O_2_ for 60 min. The cells were then allowed to recover for up to 24 h in conditioned medium, when flow cytometry based on YOPRO-1 and propidium iodide (PI) uptake evaluated cell death. In parallel, the levels of the autophagy marker beclin were probed by Western blots. In agreement with our previous data [2], significant cell death was detected in WT hTERT, while levels of YOPRO-1 and/or PI positive cells were not significantly increased in _nuc_hTERT (Figure 2A). The beclin levels were increased in a time-dependent manner in mutant-expressing cells, but they were surprisingly not augmented in cells expressing WT hTERT (Figure 2B). LC3-II levels immediately after H_2_O_2_ exposure were also increased in the mutant, but not in cells expressing WT hTERT (Figure 2C). Similarly, the phosphorylation of AMPK and decrease in the levels of phosphorylation of the mTOR downstream target p70S6 kinase (p70S6K) were robustly modulated in the mutant but not WT cells (Figure 2D–E). The parental cells (NHF) were also able to modulate the autophagy markers under oxidative stress (Figure 2F). Importantly, the activation of autophagy under oxidative stress was also reversed upon the reinstatement of hTERT to the mitochondria of mutant expressing cells (Figure 2G–I), indicating not only that autophagy was robustly upregulated in these cells under oxidative stress, but that this effect was associated to the mitochondrial, not nuclear, localization of hTERT. 

**Figure 3:**
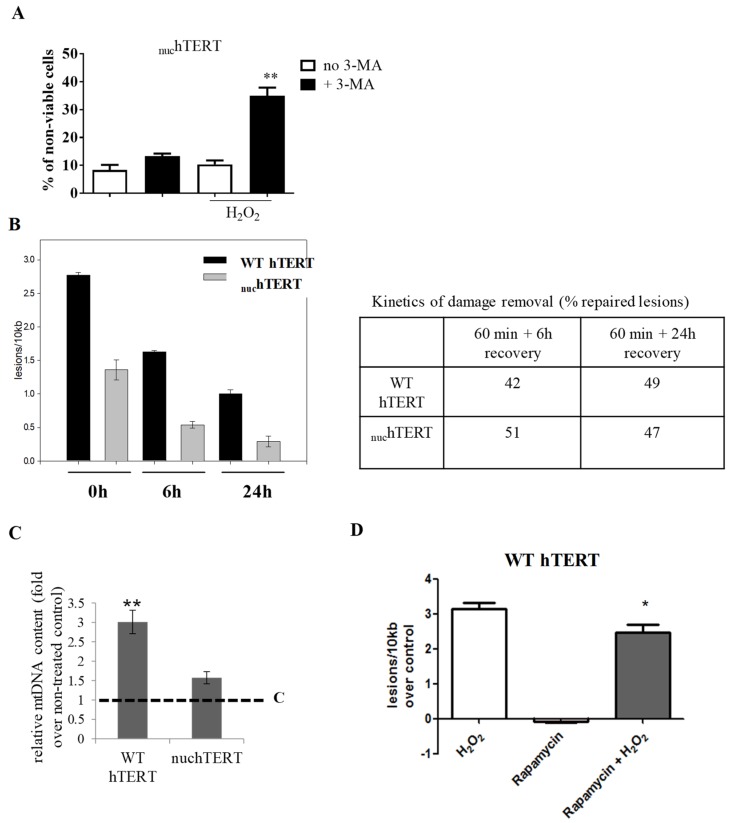
WT hTERT accumulates more mtDNA that _nuc_hTERT-expressing cells upon oxidative stress. (**A**) The _nuc_hTERT cells were pretreated with the autophagy inhibitor 3-MA for 48 h and then treated with 60 min H_2_O_2_. In addition, the cells were allowed to recover for an additional 24 h in condition media with and without the presence of 3-MA. Cell viability was determined by PI and flow cytometry. Graph represents mean of four separate biological experiments ± SEM. Student *t*-test was performed comparing the vehicle (no 3MA) + H_2_O_2_ to its respective control and 3MA + H_2_O_2_ to 3MA + no H_2_O_2_. Statistical significance was determined at a *p* value < 0.01 **. (**B**) WT and the _nuc_hTERT cells were exposed to 200 µM of H_2_O_2_ for 60 min and then allowed to recover for six and 24 h. Total genomic DNA was isolated and the mtDNA integrity was analyzed. Data was normalized to mtDNA copy number. The results represent the mean of three experiments. (**C**) Total genomic DNA was extracted from WT hTERT and _nuc_hTERT non-treated or immediately following 60 min H_2_O_2_ treatment when mtDNA content was analyzed. Graphs show average data from three independent biological experiments ± SEM. Student *t*-test compared mtDNA content at 60 min as compared to non-treated control. Statistical significance was determined at *p* value < 0.01 **. The dotted line represents non-treated control. (**D**) Quantification of mtDNA damage per 10 kb DNA by QPCR. WT hTERT expressing cells were exposed to 200 µM of H_2_O_2_ for 60 min in the presence or absence of the autophagy modulator rapamycin. The samples were pretreated for one hour with rapamycin and then exposed to H_2_O_2_ (H_2_O_2_ + rapamycin) for an additional hour. Graphs show an average from four independent experiments ± SEM. Student *t*-test compared levels of mtDNA detected in the rapamycin + H_2_O_2_ compared to H_2_O_2_ treatment alone. Statistical significance was determined at *p* value < 0.05 *. Data were normalized to mtDNA content.

Differential modulation of autophagy in nuclear- or mitochondrial hTERT-expressing cells associates with sensitivity to H_2_O_2_-induced damage. If autophagy activation underlies the resistance of _nuc_hTERT cells to H_2_O_2_-induced cell death, then its inhibition should restore the sensitivity of these cells to H_2_O_2_. As consistent with this, pre-treatment (48 h) of _nuc_hTERT cells to the autophagy inhibitor 3-methyladenine (3-MA, 10 mM), followed by treatment with 200 µM H_2_O_2_ for 60 min, led to an increase in the amount of non-viable cells at 24 h (Figure 3A).

In addition to being resistant to cell death, we also showed that the mtDNA of these cells is less sensitive to H_2_O_2-_induced damage when compared to the WT-expressing counterparts [2]. While we first proposed that this could be associated to the levels of free bioavailable iron in the cells [1], an alternative hypothesis is that mtDNA repair is slower in the WT cells; it is feasible that it is more active in the mutant, given the observed mitochondrial dysfunction at the baseline [3]. While we found that initial amount of damage is higher in the WT cells, as previously reported [2], the data presented in Figure 3B show that the kinetics of damage removal is not different between the cells. These results rule out that defects or changes in mtDNA repair *per se* underlie the different sensitivity of the mitochondrial genome of these cells to the same H_2_O_2_ challenge. 

As autophagy can aid in the removal of UV-induced mtDNA damage both in *C. elegans* and in mammalian cells in culture [12,15], the perceived “increased” mtDNA damage that was observed in WT-expressing cells may partially reflect an accumulation of damaged mtDNA due to the less robust autophagic response that was observed in these cells. At least two predictions are made by this hypothesis: (i) upon H_2_O_2_ treatment, the mtDNA content should be higher in WT hTERT cells when compared to the _nuc_hTERT counterpart; and, (ii) the promotion of autophagy should decrease mtDNA damage in WT hTERT-expressing cells. To test these, we monitored mtDNA content after H_2_O_2_ treatment by quantitative PCR, and also pharmacologically stimulated autophagy in WT cells before mtDNA damage analysis. What we found was that mtDNA content was increased in both of the cell lines upon H_2_O_2_ exposure, but that mtDNA copy number was ~2–3-fold in WT hTERT-expressing cells relative to the non-treated control or the mutant (Figure 3C). Likewise, the stimulation of autophagy by exposing WT cells to rapamycin (10 nM) for two hours prior to H_2_O_2_ exposure slightly but significantly decreased the oxidative mtDNA damage (Figure 3D). Thus, we conclude that autophagy activation plays a role in the protection that is afforded by _nuc_hTERT, both in terms of cell death and mtDNA damage. 

**Figure 4:**
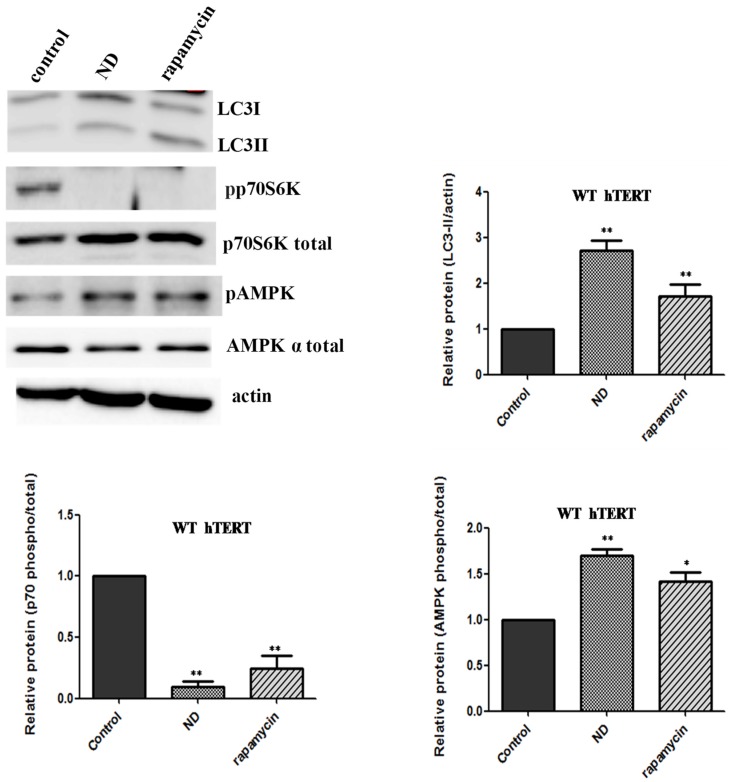
Lack of upregulation of autophagy in WT hTERT is not due to defects in signaling. The WT hTERT cells were put under conditions of nutrient deprivation (ND) or treated with 10 nM rapamycin (an mTOR inhibitor) for two hours. Autophagy activation was evaluated utilizing the LC3-II and AMPK antibodies. Phosphorylation of p70S6K (Thr 389), which is a downstream target of mTOR and classically used as a surrogate of mTOR activity, was probed. Actin was used as loading control. Graphs show average data from three independent experiments ± SEM. Student *t*-test as compared nutrient deprivation and rapamycin to untreated WT hTERT control. Statistical significance was determined at a *p* value < 0.05 * and 0.01 **.

Cells expressing WT hTERT can activate autophagy. Our data suggest that oxidative stress does not activate autophagy in cells expressing WT hTERT. One reasonable explanation for these results is that the autophagic pathway is somehow impaired in these cells. To address this, we put WT hTERT cells under the conditions of nutrient deprivation, which is a classical means of autophagy activation [16]. Under nutrient stress, autophagy is dependent on the inhibition of the kinase mTOR [17]. The cells were exposed to modified DMEM media, which lacked amino acids and serum, for two hours. In parallel cultures, the cells were also exposed to rapamycin (10 nM), which is a direct inhibitor of mTOR. The cells were collected at the end of the two-hour period and LC3-II levels, phosphorylation of p70S6K (Thr 389), and AMPK activation were assayed by immunoblots. Autophagy as judged by changes in these markers was induced in WT hTERT-expressing cells undergoing nutrient deprivation and, to a lesser extent, when exposed to rapamycin (Figure 4). The activation of autophagy was AMPK and mTOR-dependent, as the levels of phosphorylated p70S6K were significantly inhibited both under nutrient deprivation and rapamycin treatment. These data confirm that the autophagic machinery was intact in the WT hTERT cells.

**Figure 5:**
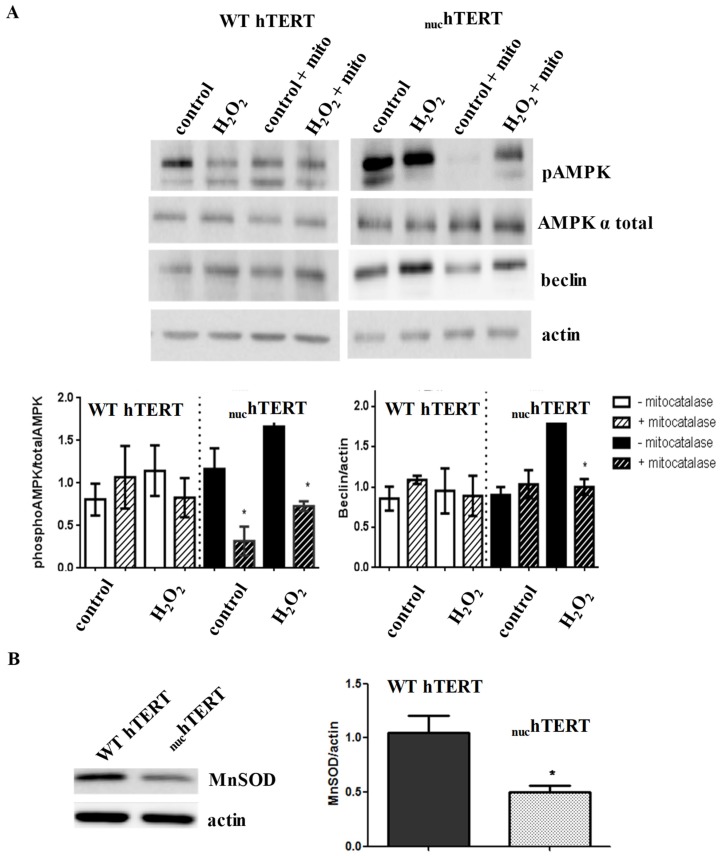
Expression of mitochondrially-targeted catalase blunts AMPK activation and autophagy in _nuc_hTERT-expressing cells. (**A**) WT hTERT and _nuc_hTERT cells were infected with an adenovirus expressing mitocatalase and 24h later cells were treated with H_2_O_2_ for 60 min. Beclin and AMPK levels were evaluated through immunoblot analysis. Actin was used as loading control. Western blots are representative; graphs are the mean of four biological experiments ± SEM. Student *t*-test compared control untreated to control mitocatalase and so forth. Statistical significance was determined at a *p* value < 0.05 *. Protein extracts were taken from untreated WT hTERT and _nuc_hTERT cells. Actin was used to normalize data. The graph represents the mean of three independent experiments. Student *t*-test compared either cell type to the untreated samples. Statistical significance was determined at a *p* value < 0.05 *. (**B**) Levels of MnSOD were assayed by immunoblots in the cells and actin was used as loading control to normalize MnSOD amounts The graph represents the mean of three independent experiments; student *t*-test compared levels in WT to mutant cells. *p* value < 0.05 *.

Mitochondrial ROS-mediates the activation of autophagy in _nuc_hTERT-expressing cells. Nutrient deprivation is known to increase the intracellular ROS levels [18], which are significantly decreased in WT hTERT-expressing cells [3]. In fact, a common feature that was reported by many about mitochondrial hTERT is its ability to significantly lower the levels of mitochondrial (and cellular) ROS [3,4,8,9,10,19]. This ability of hTERT has been demonstrated to decrease the cellular redox potential and impact ROS-dependent signaling [20]. Thus, it is possible that the decrease in mtROS that was observed in cells expressing WT hTERT may blunt the activation of autophagy under oxidative stress. Conversely, the increased in organellar ROS in the mutant cells is required for the robust autophagy response that was observed. To test this possibility, we infected WT hTERT and _nuc_hTERT cells, with an adenovirus expressing catalase that was targeted to mitochondria (mitocatalase). Catalase is a potent enzyme that is involved in the decomposition of H_2_O_2_ into H_2_O. Twenty-four hours after infection with mitocatalase, the cells were exposed to 60 min of H_2_O_2_ treatment when Western blots gauged AMPK activation and beclin levels. The results that are presented in Figure 5A show that mitocatalase had no effect on either beclin or AMPK activation in WT hTERT-expressing cells. However, it completely reversed both AMPK and beclin activation in _nuc_hTERT couterparts, reverting these cells to the WT phenotype. Taken together, these data show that the activation of autophagy in _nuc_hTERT-expressing cells is dependent on mitochondrial generated H_2_O_2_. Furthermore, the lack of autophagy activation in the WT hTERT cells is, analogously, likely due to the decreased redox potential that is associated to the presence of hTERT in the organelle. Exactly how the presence or absence of hTERT in the mitochondria modulates organellar ROS remains unclear. In trying to attain insights into this issue, we probed extracts from cells carrying the WT or mutant proteins for manganese superoxide dismutase (MnSOD), which is the mitochondrial protein that is involved in the decomposition of superoxide into H_2_O_2_. What we found was that the levels of MnSOD are significantly decreased in mutant-expressing cells (Figure 5B), which likely contributes to the increased levels of ROS in these cells. 

## 3. Discussion

Although telomerase is well studied for its role in telomere elongation, various studies have shown that hTERT impinges on the cellular response to toxicants. In the past decade, the subcellular localization of hTERT has gained special attention as a player in modulating the response to oxidative stress. Under hyperoxia and H_2_O_2_ treatment, hTERT actively accumulates in mitochondria that are seemingly dependent on its phosphorylation at tyrosine 707 by Src kinase [8,11]. It is still not known why hTERT relocates to the cytoplasm and mitochondria under oxidative stress, but it could be associated to its role in modulating mtROS production to impinge on signaling. Our current data are consistent with this hypothesis by demonstrating that the maintenance of hTERT only in the nucleus under basal and H_2_O_2_ conditions [21] leads to mtROS-dependent activation of pro-survival autophagy (Figure 2 and Figure 5), while such events are blunted in WT cells. Recently, a peptide that was encoded by telomerase RNA (TERC) was shown to play a role in the protection of drug-induced apoptosis and autophagosome formation [22]. However, since both WT and _nuc_hTERT-expressing cells express TERC, it is unlikely that this peptide plays a differential role in these cells. 

The association between hTERT, mitochondria, and ROS may serve as a means to unify discrepancies in terms of its role in protection versus sensitization to stress [1,2,8,11]. By decreasing mtROS, hTERT protects the mitochondria and perhaps the cells from endogenous oxidative damage. However, the resulting change in cellular redox state may be unfavorable to some oxidizing-dependent signaling pathways, while potentially favoring reducing reactions. For instance, hTERT was shown to alter the cellular response to TNFα through a redox mechanism [20]. Our data is in line with these findings, as indicated by the fact that AMPK activation of autophagy is dampened in cells expressing WT hTERT or upon mitocatalase expression. AMPK has been shown to be directly activated by ROS [14], as have been the other Atg genes that are involved in the autophagic response [23]. In addition, other findings show that ROS are required to induce autophagy under starvation conditions [24]. Although the target(s) that are affected by the changes in mitochondrial redox state upon the expression of hTERT in mitochondria remain unknown, our data place mitochondrial H_2_O_2_ as an important signaling molecule that modulates autophagy based on the hTERT’s subcellular localization. Why exactly the absence of hTERT in mitochondria leads to increased mitochondrial ROS remains unknown, but our data would suggest that decreased levels of MnSOD might play a role. In turn, this could increase the oxidative damage within the organelle and lead to changes in the electron transfer efficiency, overall contributing to leakage at the ETC. It will be important to assay MnSOD activity in these cells to define whether the decreased levels of the protein indeed impart effects on the overall mitochondrial ROS. Most notably, understanding why and how the levels of MnSOD change based on the subcellular localization of hTERT may lead to important insights regarding the crosstalk between telomerase, mitochondria, and cellular redox changes. 

Accumulating evidence suggests that the outcomes of autophagy modulation, including those that are mediated by ROS, can either promote cell survival or may be associated with cell death [25]. Decreased autophagy may provide a cellular environment allowing for the accumulation of dysfunctional mitochondria [26], perhaps channeling the cells to apoptosis. We had previously proposed that the increased levels of apoptosis that were caused when hTERT was mitochondrial were a means to cull out the dysfunctional organelles (and cells) from the population [1]. It is possible that the impaired autophagic clearance of damaged organelles that are caused by mitochondrial hTERT observed here could be the means through which the accumulation of damaged organelles causes the cells to die. Conversely, the strong activation of autophagy in _nuc_hTERT mutant-expressing cells leads to resistance to H_2_O_2_-induced cell death, despite dysfunctional mitochondria. These results may help to explain recent findings, in which the extinction of TERT in a cancer prone mouse model initially led to tumor reversal, which was associated to short telomeres and mitochondrial dysfunction, but later to the re-emergence of a resistant population. The latter had re-established telomere maintenance through ALT but, interestingly, mitochondrial function and ROS production were not fully restored [7]. While the authors concluded that telomere dysfunction facilitated resistance amongst the reemerging tumors [7], one alternative explanation is that this effect may be mitochondrial-driven. It is possible that the initial loss of cellular telomerase caused telomere dysfunction and set in motion a mitochondrially-driven cascade. In our study, this cascade involved the adaptation by activation of autophagy, a pro-survival mechanism that is shown to be upregulated in some cancers [27]. It would be interesting to evaluate autophagy activation in these re-emergent tumors. 

## 4. Materials and Methods 

### 4.1. Cells and Cell Culture

Normal human fibroblasts (NHF) stably expressing WT hTERT and _nuc_hTERT have been previously described [2]. To reinstate the mitochondrial pool of hTERT, _nuc_hTERT -expressing cells were infected with full length WT hTERT, as expressed in a retroviral vector (hTERTpLXIN). Infections were performed following our previous work [2] and the cells expressing the two vectors were selected based on double resistance of puromycin (_nuc_hTERT pBabe) and G418 (hTERTpLXIN). The cell lines were cultured in DMEM/F-12 medium (ThermoFisher Invitrogen, Grand Island, NY, USA), supplemented with 10% fetal bovine serum, 1% penicillin/streptomycin, and 2 μg/mL of puromycin and/or 800 µg/mL of G418. Cells were maintained in 5% CO_2_ at 37 °C. The cells were subcultured approximately every three days or sooner if needed.

### 4.2. H_2_O_2_ Treatments

Hydrogen peroxide (H_2_O_2_) (Millipore Sigma, St. Louis, MO, USA) treatments were performed, as described [28]. Briefly, the cells were challenged with 200 μM H_2_O_2_ for 60 min and then harvested immediately after treatment upon washing with (1X) phosphate buffer saline (PBS, ThermoFisher Invitrogen, Grand Island, NY, USA). For all experiments, the cells were seeded 15–18 h prior to the experiments. Immediately before treatment, the cells were washed once with DMEM/F-12 (ThermoFisher Invitrogen, Grand Island, NY, USA) without any supplements, and the conditioned medium was saved for later use for studies where recovery was performed.

### 4.3. Cell Viability

For autophagy inhibition, NHF _nuc_hTERT were exposed to a final concentration of 10 mM 3-MA (Millipore Sigma, St. Louis, MO, USA) for 48 h, prior to treatment with H_2_O_2_. After the H_2_O_2_ exposure cells were washed and allowed to recover in conditioned media for an additional 24 h in the presence and absence of 3-MA. Cell viability was analyzed by PI exclusion by flow cytometry as well as using trypan blue exclusion. The cells were evaluated both immediately after the treatments, as well as upon the 24 h of recovery from H_2_O_2_ exposure.

### 4.4. Apoptosis

Cells were seeded 15–18 h prior to subsequent treatments. WT hTERT and _nuc_hTERT were submitted to 60 min of 200 µM H_2_O_2_ treatment. Cells were allowed to recover in conditioned medium for 24 h. The cells were then harvested and then assayed with YOPRO-1 and PI to monitor apoptosis (YOPRO-1, ThermoFisher Invitrogen, Grand Island, NY, USA ) and cell death (PI) using flow cytometry. In brief, after trysinization, the cells were washed twice with 1 mL of PBS, resuspended in PBS, and then stained with a final concentration of 2.5 µM of YO-PRO-1 (ThermoFisher Invitrogen, Grand Island, NY, USA) and 1 µg of PI (ThermoFisher Invitrogen, Grand Island, NY, USA) for 20 min on ice. Cells were then scored as viable (negative for both markers), apoptotic YOPRO-1 positive/PI-negative or YOPRO-1 positive/PI-positive, and dead (PI positive) while using a BD Biosciences FACS Calibur flow cytometer.

### 4.5. Nutrient Deprivation and Rapamycin Exposure

For nutrient deprivation, NHF WT hTERT were maintained for two hours in modified DMEM media composed of: 138 mM NaCl, 1X DMEM salts, 24 mM NaHCO_3_, 1.8 mM CaCl_2_, 10 mM glucose, 0.3 mM pyruvate, Vitamin mixture, 40 mg/mL Fe(NO_3_)_3_9H_2_O, 1% penicillin/strep, 50 μg/L gentamycin lacking amino acids, and serum. The cells were also exposed to rapamycin (10 nM) for two hours. The time for nutrient deprivation and rapamycin treatment were selected based on time course and dose response experiments monitoring beclin and LC3-II levels by immunoblot as readouts of autophagy induction (not shown). 

### 4.6. Western Blot Analysis

The protein extracts were prepared from independent cultures by resuspension in 1X Chaps Lysis Buffer containing protease inhibitor cocktail (Millipore Sigma, St. Louis, MO, USA) and phosphatase arrest III (Biosciences). Lysates were centrifuged and supernatant was recovered. Protein content was then estimated using the Lowry Assay. Proteins were separated on 4%, 10%, 12%, or 15% SDS-PAGE gels, and in some cases in 4–12% precast gradient gel (Novex; ThermoFisher Invitrogen, Grand Island, NY, USA), and then blotted onto nitrocellulose membranes. The membranes were blocked with 5% nonfat milk in PBS-T (phosphate buffered with 0.05%-Tween 20) at room temperature for one hour and subsequently probed with primary antibodies. The following antibodies were used: BECN1 (beclin 1) (Santa Cruz Biotechnology, Santa Cruz, CA, USA); LC3 (Novus biologicals, Centennial, CO, USA); phospho-p70S6 kinase (Thr389) (Cell Signaling, Danvers, MA, USA); p70S6 kinase (Cell Signaling, Danvers, MA, USA); phospho-AMPKα (Thr172) (Cell Signaling, Danvers, MA, USA); AMPKα (Cell Signaling, Danvers, MA, USA); and, Actin (Millipore Sigma, St. Louis, MO, USA) were used as a loading control. All of the primary antibodies were diluted to 1:1000, unless otherwise specified and the respective secondary antibodies were used in a 1:10,000 dilution. The protein bands were visualized by using the Amersham ECL detection system (GE Healthcare, Glenn Allen, VA, USA) or Femto Maximum Sensitivity Substrate (ThermoFisher, Grand Island, NY, USA) with the operation of the Fujifilm multi-gauge V3.1 software.

### 4.7. Adenovirus Infection

Adenovirus expressing mitochondria-targeted catalase was obtained as a gift from Dr. Marcelo Bonini from the University of Illinois at Chicago. WT hTERT and _nuc_hTERT cells were plated in DMEM/F-12 (ThermoFisher Invitrogen, Grand Island, NY, USA) medium with 10% Fetal bovine serum and then allowed to attach to six-well plate dishes overnight at 37 °C before the desired amount of viral particles were added. The next day, cells were washed in warm PBS (2X) and 500 µL DMEM/F-12 serum free medium were added to each well. 1 µL mito-catalase adenovirus was subsequently added to each well and incubated for one-hour at 37 °C, swirling every 15 min. Fresh medium with serum was added the following day. On the following day, the cells were treated with and without 200 μM H_2_O_2_ for 60 min. The samples were then collected for western analysis.

### 4.8. Mitochondria Isolation

The harvested cells were washed twice with PBS, centrifuged (1000 g, minutes), and the supernatants were discarded. The pellets were weighed and gently resuspended in HEPES dissociation buffer (5 mM KPO4, pH 7.5, 2 mM KCl, 1 mM 2-mercaptoethanol), at 9× volume of the wet weight of the cells. Protease inhibitor cocktail (1:100 v/v) was added, and the cells were left on ice for one hour. Once the cells were swollen and they were broken by dounce homogenization for 20–30 strokes until only 0–2 intact cells/microscope field remained. Sucrose buffer 2.5 × (0.625 M sucrose, 20 mM Tris–HCl, 5 mM EGTA, 5 mM KCl, pH 7.5) was added to a 1 × concentration. To collect nuclei, the cells were centrifuged for five minutes at 600 g (4 °C). The supernatants with remaining mitochondria were collected, while the pellet was kept as the nuclear fraction. This centrifugation step was repeated to make sure the broken or unbroken cells nuclei were completely pelleted. Supernatants were then centrifuged for 30 min at 12,000 g; the pellets containing mitochondria were washed three times with 1 × 0.25 M sucrose buffer to remove the remaining nuclear DNA and proteins, and then treated with proteinase K (10 µg mL^−1^ final concentration) for 30 min on ice. Protease inhibitors 10 µL of (PMSF 100 mM) were added, and samples were incubated on ice for an additional 10 min. Mitochondrial pellet was then washed three times with 1× sucrose buffer (20 min at 12,000 g). The mitochondrial pellets were resuspended in lysis buffer (4% SDS, 0.3 M NaCl, 10% glycerol, 20 mM Tris–HCl, pH 8.0, 14 mM 2-mercaptoethanol, and proteinase inhibitors) and left on ice for 30 min. The protein was quantified using the Lowry method. Equal amounts of 40 µg lysed whole cell and mitochondria prep samples from each cell lines were run on 4–12% gradient gel. 

### 4.9. DNA Damage and DNA Isolation 

Kinetics of mtDNA repair was followed using gene-specific quantitative PCR (QPCR) measuring restoration of amplification of the target DNA after the removal of H_2_O_2_, as described [28]. The cells were treated with H_2_O_2_ for one hour and were either harvested immediately (time 0) or they could recover in conditioned medium for six or 24 h. MtDNA damage was also estimated in the cells upon treatment with the autophagy modulator rapamycin, as described above. For DNA damage analysis, the total genomic DNA was isolated and the integrity of the mtDNA was measured using two sets of primers to the mtDNA. Specific primers were used to amplify a large 8.9-kb fragment of human mtDNA, to determine mtDNA integrity, and a small 221-bp fragment to monitor the mtDNA copy number. The latter was used to normalize the data that were obtained with the large fragment [28]. Relative amplifications were calculated and used to assess the damage frequency per 10 kb of the genome, assuming a Poisson distribution of damage on the template. This assay is centered on the fact that DNA lesions block/slow the progression of the polymerase, so that only undamaged templates take part in the PCR reaction. Therefore, the amplification is inversely relative to DNA damage or in other words the more lesions on the target DNA, the less amplification. 

### 4.10. Statistical Analysis

Significance was calculated using Student’s *t*-test with GraphPad Prism software. Data were considered to be statistically significant when the probability value (*p*) was less than 0.05 * and 0.01 **. Values are represented as average ± S.E.M.

## Abbreviations

3-MA	3-methyladenine
AMPK	AMP-activated protein kinase
H_2_O_2_	hydrogen peroxide
hTERT	human telomerase reverse transcriptase
hTR	human telomerase RNA
mtDNA	mitochondrial DNA
OXPHOS	oxidative phosphorylation
PI	propidium iodide
ROS	reactive oxygen species
